# Fgfr4 combined with inflammatory cytokines improves recurrence risk prediction in differentiated thyroid carcinoma: A retrospective cohort study

**DOI:** 10.5937/jomb0-59084

**Published:** 2026-01-06

**Authors:** Yongjie Hu, Peifei Huang, Yingying Sha

**Affiliations:** 1 Shanghai University of Traditional Chinese Medicine, Department of General Surgery, Yueyang Hospital of Integrated Traditional Chinese and Western Medicine, Shanghai, 200437, China

**Keywords:** FGFR4, differentiated thyroid cancer, inflammatory factors, diagnosis, recurrence, FGFR4, diferencirani rak štitne žlezde, inflamatorni faktori, dijagnoza, recidivi

## Abstract

**Background:**

This study aimed to evaluate the synergistic role of fibroblast growth factor receptor 4 (FGFR4) and inflammatory cytokines (ICs) (interleukin-6 [IL-6], tumor necrosis factor-a [TNF-a], and C-reactive protein [CRP]) in predicting recurrence of differentiated thyroid carcinoma (DTC) after radical surgery, and to develop a combined predictive model for improved postoperative risk stratification.

**Methods:**

We enrolled 102 DTC patients treated between February 2022 and January 2024, along with 98 healthy controls. Serum levels of FGFR4, IL-6, TNF-a, and CRP were measured preoperatively and postoperatively using ELISA. Independent risk factors were identified through logistic regression, diagnostic performance was assessed using ROC analysis, and correlations of FGFR4 and ICs with postoperative recurrence were evaluated.

**Results:**

Preoperative levels of FGFR4, IL-6, TNF-a, and CRP were significantly elevated in DTC patients compared to healthy controls (P&lt;0.05). A diagnostic model integrating these four markers demonstrated superior performance (AUC=0.931; sensitivity 94.12%, specificity 79.59%) over individual biomarkers (P&lt;0.05). Among DTC patients, those with recurrence (n = 26) exhibited significantly higher FGFR4 and inflammatory cytokine levels than the non-recurrent group (P&lt;0.05). The combined model predicted 1-year recurrence with an AUC of 0.864 (sensitivity 73.08%, specificity 93.42%).

**Conclusions:**

The synergistic interaction between FGFR4 and ICs plays a critical role in DTC. Their combined detection enhances postoperative recurrence risk prediction, offering a valuable tool for clinical risk stratification.

## Abstract (sr)

**Uvod:**

Cilj ove studije bio je da se proceni sinergistička uloga receptora 4 faktora rasta fibroblasta (FGFR4) i inflamatornih citokina (IC) (interleukin-6 [IL-6], faktor nekroze tumora-a [TNF-a] i C-reaktivni protein [CRP]) u predviđanju recidiva diferenciranog karcinoma štitne žlezde (DTC) nakon radikalne operacije, i da se razvije kombinovani prediktivni model za poboljšanu postoperativnu stratifikaciju rizika.

**Metode:**

Uključili smo 102 pacijenta sa DTC lečenih između februara 2022. i januara 2024. godine, zajedno sa 98 zdravih kontrola. Serumski nivoi FGFR4, IL-6, TNF-a i CRP mereni su preoperativno i postoperativno pomoću ELISA testa. Nezavisni faktori rizika identifikovani su logističkom regresijom, dijagnostičke performanse su procenjene pomoću ROC analize, a procenjene su korelacije FGFR4 i IC sa postoperativnim recidivom.

**Rezultati:**

Preoperativni nivoi FGFR4, IL-6, TNF-a i CRP bili su značajno povišeni kod pacijenata sa DTC u poređenju sa zdravim kontrolama (P&lt;0,05). Dijagnostički model koji integriše ova četiri markera pokazao je superiorne performanse (AUC=0,931; osetljivost 94,12%, specifičnost 79,59%) u odnosu na pojedinačne biomarkere (P&lt;0,05). Među pacijentima sa DTC, oni sa recidivom (n = 26) pokazali su značajno više nivoe FGFR4 i inflamatornih citokina nego grupa sa nerecidivom (P&lt;0,05). Kombinovani model je predvideo recidiv nakon 1 godine sa AUC od 0,864 (senzitivnost 73,08%, specifičnost 93,42%).

**Zaključak:**

Sinergistička interakcija između FGFR4 i IC igra ključnu ulogu u DTC. Njihova kombinovana detekcija poboljšava predviđanje rizika od postoperativnog recidiva, nudeći vredan alat za stratifikaciju kliničkog rizika.

## Introduction

Differentiated thyroid carcinoma (DTC), predominantly comprising papillary and follicular subtypes, represents the most prevalent malignancy of the endocrine system [1]. While the majority of DTC patients exhibit favorable prognoses following surgical intervention combined with radioactive iodine therapy, approximately 10-30% develop local recurrence or distant metastasis after radical resection, presenting a substantial clinical challenge for long-term survival and quality of life [2]. Current postoperative recurrence risk assessment predominantly relies on TNM staging, serum thyroglobulin levels, and imaging modalities [3]. However, these conventional diagnostic parameters demonstrate limited sensitivity and specificity in predicting early recurrence risk and fail to elucidate the underlying molecular mechanisms driving tumor recurrence [4]. Consequently, there exists a pressing need to identify novel biomarkers to optimize recurrence risk stratification systems, thereby facilitating personalized therapeutic strategies and improving clinical outcomes.

In recent years, the tumor microenvironment has emerged as a critical focus in cancer research, with growing recognition of its pivotal role in tumor progression. Among its key components, the fibroblast growth factor receptor (FGFR) family has been strongly implicated in regulating tumor cell proliferation, invasion, and metastatic potential [5]. FGFR4, as a prominent member of this family, has been demonstrated to drive malignant progression in various solid tumors through activation of crucial signaling pathways such as MAPK/ERK and PI3K/AKT [6] [7]. Despite these advances, the expression profile of FGFR4 in DTC and its clinical relevance to postoperative recurrence remain poorly characterized and require systematic investigation. Concurrently, inflammatory responses—a hallmark feature of the tumor microenvironment—have been shown to facilitate tumor recurrence through angiogenesis induction and immune evasion mediated by pro-ICs such as interleukin-6 (IL-6), tumor necrosis factor-α (TNF-α), and C-reactive protein (CRP) [8]. Intriguingly, emerging evidence suggests a potential crosstalk between FGFR signaling pathways and inflammatory cytokine networks [9], yet this interaction and its functional significance in DTC recurrence remain completely unexplored. We postulate that combining FGFR4 expression analysis with inflammatory cytokine profiling could overcome the limitations of single-biomarker approaches, thereby offering a more robust, multidimensional framework for recurrence risk assessment.

To test this hypothesis, our study will pioneer a comprehensive approach by integrating FGFR4 expression patterns with systemic inflammatory signatures to develop a novel predictive model for postoperative DTC recurrence. Through multifaceted analysis combining molecular biology assays with longitudinal clinical data, we aim to: (1) delineate the synergistic mechanisms between FGFR4 signaling and inflammatory microenvironment dynamics, and (2) validate their combined utility as early-warning biomarkers for recurrence. This investigation not only promises to advance fundamental understanding of DTC recurrence mechanisms but may also yield significant clinical benefits by enabling personalized therapeutic strategies and improving long-term patient outcomes.

## Materials and methods

### Study subjects

**Figure 1:**
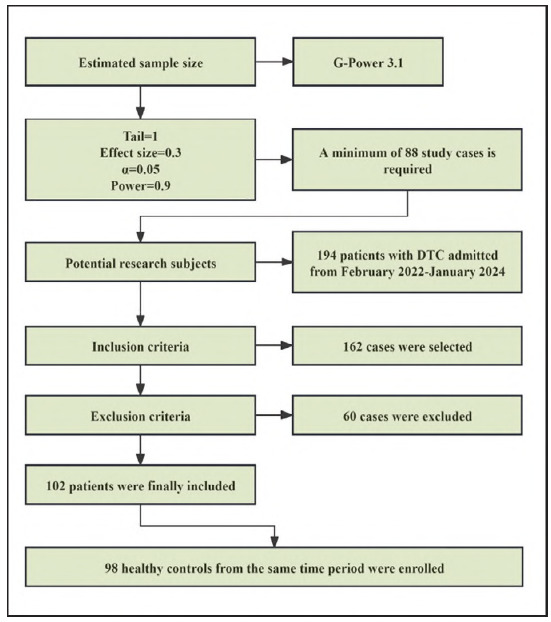
Screening process for research subjects.

This study enrolled 102 DTC patients admitted between February 2022 and January 2024, along with 98 healthy individuals who underwent routine physical examinations during the same period. The screening process for participant selection is summarized in Figure 1. DTC patients were 26 males and 76 females with age (56.08±6.70) years and duration of disease (6.69±2.43) months. Physical examiners were 30 males and 68 females aged (55.19± 5.45) years. The study protocol was approved by the Institutional Ethics Committee (NO. KL2022213), and written informed consent was obtained from all participants. The research adhered to the principles of the Declaration of Helsinki, and participants were blinded to group allocation.

Inclusion Criteria: Histopathologically confirmed DTC diagnosis; Radical thyroidectomy performed by a standardized surgical team; Availability of complete clinical records, including preserved tissue and serum samples.

Exclusion Criteria: History of other malignancies or synchronous secondary primary tumors: Active infections, autoimmune disorders, or long-term immunosuppressive therapy; Loss to follow-up or incomplete postoperative clinical data; High-grade or aggressive DTC variants; Known hereditary thyroid cancer syndromes; Mortality during treatment or follow-up.

### Prognostic follow-up

All DTC patients underwent prognostic follow-up for a minimum of one year, which involved regular re-examinations. During the first six months, patients were required to attend follow-up examinations at least once per month. Subsequently, follow-up visits were scheduled at least once every two months. Any instances of DTC recurrence were documented throughout the follow-up period.

### Detection methods

Serum samples were collected preoperatively after an overnight fast and postoperatively at 6 weeks (±3 days) during follow-up visits. Serum thyroid hormone levels were measured using a fully automated chemiluminescence immunoassay analyzer. Additionally, enzyme-linked immunosorbent assay (ELISA) was employed to quantify serum levels of FGFR4, IL-6, TNF-α, and CRP Commercial ELISA kits were utilized for these assays (FGFR4: Cusabio, CSB-CF008648HU; IL-6 and TNF-α: R&D Systems, 2305-FL-025/CF and AFL210; CRP: Abcam high-sensitivity kit, ab260058). Standard concentration gradients were prepared in strict accordance with the manufacturer's protocols. The 96-well plates were coated with capture antibodies and incubated overnight at 4°C. Following this, the plates were blocked with 1% bovine serum albumin (BSA). Subsequently, serum samples, biotinylated detection antibodies, and streptavidin-conjugated horseradish peroxidase (HRP) were sequentially added to the wells. Each step was followed by incubation at 37°C and thorough washing to remove unbound substances. Color development was initiated by adding 3,3',5,5'-tetramethylbenzidine (TMB) substrate, and the reaction was allowed to proceed for 15 minutes before being terminated with 2 mol/L sulfuric acid (H_2_SO_4_). Absorbance was measured at dual wavelengths of 450 nm and 570 nm to ensure accuracy. A four-parameter logistic curve was generated from the standard curve to determine analyte concentrations. Only samples with an inter-assay coefficient of variation (CV) of less than 10% between duplicates were considered valid; samples exceeding this threshold were reanalyzed [10].

### Observation indicators

This study examined the differential expression levels of FGFR4, IL-6, TNF-α, and CRP levels between DTC patients and healthy controls. Additionally, the diagnostic potential of pre-treatment FGFR4, IL-6, TNF-α, and CRP levels for DTC detection was assessed, along with the efficacy of their post-treatment levels in predicting prognostic recurrence (the presence of any clinical manifestation of thyroid cancer confirmed by clinical and imaging examination was defined as DTC recurrence). Recurrence assessment was performed by clinicians who were aware of patients' clinical data. Potential bias was mitigated by cross-validation with imaging and serum biomarkers.

### Statistical methods

Data analysis was performed using SPSS 26.0. Continuous variables with normal distribution (confirmed by Shapiro-Wilk testing) were expressed as mean ± standard deviation (SD) and analyzed via independent t-tests. Paired t test was used to compare the data before and after treatment. Categorical variables were presented as frequencies [n (%)] and compared using chi-square tests. Pearson's correlation was employed to assess variable associations. For combined biomarker evaluation, logistic regression modeling generated a composite predictive equation, with subsequent receiver operating characteristic (ROC) curve construction. Diagnostic performance was quantified through ROC analysis, including area under the curve (AUC) calculations.

## Results

### Expression levels of FGFR4 and ICs

**Figure 2:**
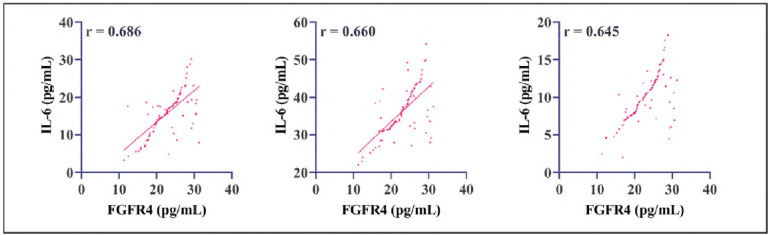
Correlation analysis of FGFR4 and inflammatory factors.

Pretreatment DTC patients demonstrated significantly higher serum levels of FGFR4, IL-6, TNF-α, and CRP compared to healthy controls (P<0.05). Logistic regression analysis identified FGFR4, IL-6, TNF-α, and CRP as independent risk factors for DTC (P<0.05, Table 1), implicating their potential roles in the pathogenesis and progression of DTC. Furthermore, Pearson correlation analysis revealed significant positive associations between FGFR4 expression and the levels of IL-6, TNF-α, and CRP in DTC patients (P<0.05, Figure 2), suggesting a possible interplay between FGFR4 signaling and inflammatory pathways in DTC development.

**Table 1:** Comparison of clinical data of control group and PTC patients.

Groups	n	FGFR4 (pg/mL)	IL-6 (pg/mL)	TNF-α (pg/mL)	CRP (mg/L)
Healthy controls	98	16.54±5.94	11.94±5.05	30.50±6.71	6.57±2.00
DTC patients	102	22.98±4.45	15.89±5.51	36.25±6.34	9.89±3.09
t		8.699	5.276	6.224	8.977
P		<0.001	<0.001	<0.001	<0.001
Factor	Regression<br>coefficient	Standard<br>error	Wals	P	OR (95%CI)
FGFR4	0.235	0.049	22.979	<0.001	1.265 (1.149-1.393)
IL-6	0.155	0.044	12.369	<0.001	1.168 (1.071-1.273)
TNF-α	0.123	0.36	11.460	0.001	1.131 (1.053-1.214)
CRP	0.489	0.100	24.001	<0.001	1.631 (1.341-1.984)
Constant	-14.835	2.153	47.489	<0.001	

### Diagnostic efficacy of FGFR4 and ICs in DTC

**Figure 3:**
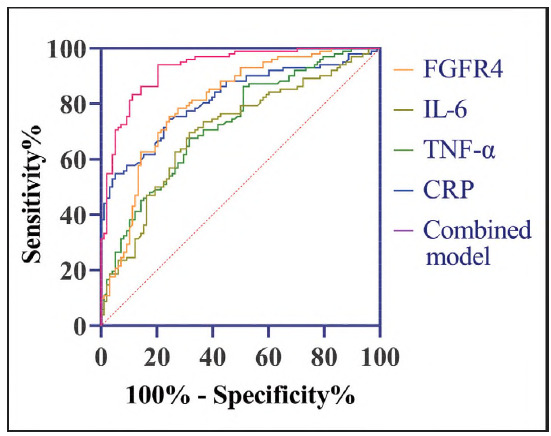
ROC curves of FGFR4 and inflammatory factors for the diagnosis of DTC.

ROC curve analysis revealed that FGFR4, IL-6, TNF-α, and CRP each demonstrated strong diagnostic performance for detecting DTC (P<0.05). Notably, the combination of these four markers achieved a sensitivity of 94.12% and a specificity of 79.59% (P<0.05, Figure 3 and Table 2), with an AUC of 0.931. This integrated model outperformed individual biomarkers, offering superior diagnostic accuracy and clinical utility.

**Table 2:** Effectiveness of FGFR4 and inflammatory factors in diagnosing DTC.

Diagnostic methods	Cut-off	AUC	Sensitivity (%)	Specificity (%)	P
FGFR4	>19.95 pg/mL	0.805	75.49	75.51	<0.001
IL-6	>14.18 pg/mL	0.703	69.61	68.37	<0.001
TNF-α	>32.97 pg/mL	0.728	67.65	68.37	<0.001
CRP	>7.83 pg/mL	0.818	73.53	76.53	<0.001
Combined model	>0.385	0.931	94.12	79.59	<0.001

### Association of FGFR4 and ICs with DTC prognostic recurrence

Post-treatment levels of FGFR4, IL-6, TNF-α, and CRP were significantly reduced compared to baseline values (P<0.05). During the follow-up period, 26 patients had recurrence, and the total recurrence rate was 25.49%. Compared to non-recurrent cases, those with recurrence exhibited markedly elevated post-treatment levels of these biomarkers (P<0.05). Similarly, logistic regression analysis confirmed that high post-treatment expression of FGFR4, IL-6, TNF-α, and CRP served as independent risk factors for DTC recurrence (P<0.05, Table 3).

**Table 3:** Relationship between FGFR4, inflammatory factors and prognostic recurrence of DTC.

Groups	n	FGFR4 (pg/mL)	IL-6 (pg/mL)	TNF-α (pg/mL)	CRP (mg/L)
Before treatment	102	22.98±4.45	15.89±5.51	36.25±6.34	9.89±3.09
After treatment	102	19.94±3.77	14.43±3.30	32.47±5.41	7.48±3.30
t		10.483	2.302	4.577	5.383
P		<0.001	0.022	<0.001	<0.001
Groups	n	FGFR4 (pg/mL)	IL-6 (pg/mL)	TNF-α (pg/mL)	CRP (mg/L)
Non-recurrent	76	16.20±3.11	13.78±2.78	31.47±5.44	6.78±2.94
Recurrence	26	19.10±4.67	16.33±3.95	35.41±4.20	9.51±3.51
t		3.528	3.601	3.365	3.885
P		<0.001	<0.001	0.001	<0.001
Factors	Regression<br>coefficient	Standard<br>error	Wals	P	OR<br>(95%CI)
FGFR4	0.240	0.097	6.166	0.013	1.272 (1.052-1.538)
IL-6	0.248	0.108	5.256	0.022	1.281 (1.037-1.584)
TNF-α	0.126	0.060	4.333	0.037	1.134 (1.007-1.227)
CRP	0.361	0.111	10.604	0.001	1.435 (1.115-1.783)
Constant	-16.114	3.455	21.757	<0.001	-

### Prognostic value of FGFR4 and ICs in predicting DTC recurrence

**Figure 4:**
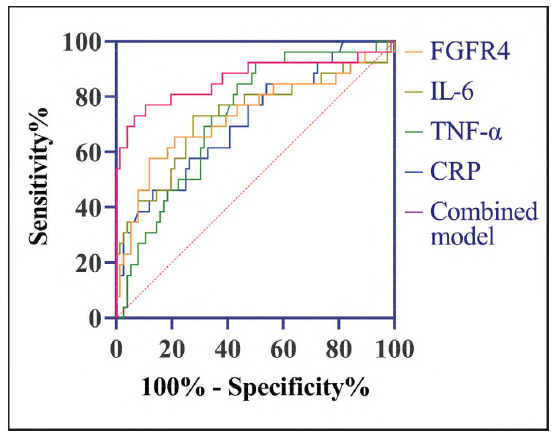
ROC curves for prognostic recurrence of DTC diagnosed by FGFR4 and inflammatory factors.

ROC curve analysis also highlighted the robust prognostic capability of the combined model (FGFR4+IL-6 + TNF-α + CRP). For predicting one-year DTC recurrence, the model yielded a sensitivity of 73.08% and a specificity of 93.42% (P<0.05, Figure 4 and Table 4), with an AUC of 0.864, again surpassing the performance of individual markers.

**Table 4:** Effectiveness of FGFR4 and inflammatory factors in diagnosing prognostic recurrence of DTC.

Diagnostic methods	Cut-off	AUC	Sensitivity (%)	Specificity (%)	P
FGFR4	>19.62 pg/mL	0.731	57.69	88.16	<0.001
IL-6	>15.48 pg/mL	0.731	73.08	72.37	<0.001
TNF-α	>31.13 pg/mL	0.730	92.31	50.00	<0.001
CRP	>10.33 pg/mL	0.714	46.15	86.84	<0.001
Combined model	>0.322	0.864	73.08	93.42	<0.001

## Discussion

This study found that FGFR4, IL-6, TNF-α and CRP were increased in DTC patients, and showed excellent predictive effects on the prognosis and recurrence of DTC. These findings provide a more reliable safety guarantee for the treatment of DTC in the future.

The study revealed that preoperative FGFR4 levels in DTC patients were significantly elevated compared to healthy individuals and positively correlated with recurrence risk. This suggests that FGFR4 may facilitate the reactivation of residual lesions post-surgery by modulating tumor cell survival pathways. As a member of the FGFR family, FGFR4 has been extensively implicated in tumor progression through multiple mechanisms: (1) Promoting tumor cell pro-liferation and anti-apoptosis: Upon activation, FGFR4 phosphorylates transcription factors such as c-Myc and Cyclin D1 via the MAPK/ERK pathway, accelerating cell cycle progression [11] [12]. Concurrently, activation of PI3K/AKT pathway suppresses pro-apoptotic proteins, enhancing tumor cell survival [13]. (2) Driving epithelial-mesenchymal transition (EMT) and metastasis: By upregulating EMT-related transcription factors and reducing E-cadherin expression, FGFR4 signaling enhances tumor cell invasiveness [14] [15]. (3) Regulating tumor metabolic reprogramming: The study by D'Agosto S et al. demonstrated that FGFR4 activates the mTORC1 pathway, promoting glycolysis and glutamine metabolism to supply the energy and macromolecules necessary for rapid tumor proliferation [16]. This metabolic adaptability may be crucial for the survival of residual lesions post-surgery, further supporting the potential of FGFR4 as a biomarker for assessing DTC recurrence risk. Following treatment, FGFR4 levels decreased but remained elevated in patients with recurrence, indicating its persistent oncogenic role in the recurrence microenvironment. This observation is consistent with studies in breast cancer, where high FGFR4 expression correlates with poor prognosis [17], suggesting that FGFR4 may serve as a therapeutic target across multiple cancer types.

As previously noted, abnormal FGFR4 expression has been implicated in various malignancies, suggesting that relying solely on FGFR4 as a biomarker for evaluating DTC progression may lack diagnostic specificity. This study further supports this notion, demonstrating that FGFR4 alone exhibited a sensitivity of 94.12% and a specificity of 79.59% in diagnosing DTC occurrence. To enhance the diagnostic and prognostic utility of FGFR4, we explored its combination with complementary biomarkers. It is well established that the inflammatory tumor microenvironment plays a critical role in promoting angiogenesis and immune evasion through pro-ICs such as IL-6 and TNF-α, which have been linked to tumor recurrence in multiple cancers [18]. Our findings revealed elevated levels of these inflammatory mediators in DTC patients, showing a significant positive correlation with FGFR4 expression. Mechanistically, IL-6 and TNF-α may upregulate FGFR4 via NF-B pathway activation, while FGFR4 signaling, in turn, amplifies inflammatory cytokine release [19] [20]. This bidirectional interaction forms a self-reinforcing loop that drives tumor proliferation and immune escape, offering novel insights into the molecular mechanisms underlying DTC recurrence. Integrating FGFR4 with ICs significantly enhanced diagnostic accuracy for both DTC detection and recurrence prediction. This multi-parameter approach captures the complex interplay between tumor biology and host immune responses, enabling more comprehensive risk stratification. Such a strategy could facilitate early identification of high-risk patients, guiding timely clinical intervention. The AUC of each index was lower than that of the combined model, which was also due to the fact that a variety of life activities in the human body may lead to abnormal changes in inflammatory response, which also emphasized the necessity of the combined model. Similar multi-marker models have been successfully applied in thyroid cancer, where biomarker combinations have substantially enhanced prognostic precision [21].

This study presents several notable strengths. To our knowledge, it is the first to integrate FGFR4 expression with systemic inflammatory cytokine profiles to construct a comprehensive predictive model for DTC recurrence. The use of stringent inclusion and exclusion criteria further enhances the reliability of our findings by minimizing confounding bias. Building on these results, we propose three key translational directions: (1) development of a multiplex detection kit for FGFR4 and ICs to facilitate postoperative recurrence risk stratification; (2) FGFR4 inhibition may synergize with anti-inflammatory therapies, though preclinical validation is needed; and (3) establishment of a dynamic monitoring system to assess therapeutic efficacy by serial measurement of biomarker levels before and after intervention. However, several limitations should also be acknowledged. First, the relatively small sample size and single-center retrospective design may affect the generalizability of our findings, underscoring the need for validation through multicenter prospective studies with larger cohorts. Second, the follow-up duration was insufficient to capture long-term recurrence events, potentially leading to an underestimation of recurrence rates. Third, due to limited foundational data, this study did not explore the potential influence of FGFR4 gene mutations or epigenetic modifications on its functional role. Future research should incorporate multi-omics approaches to elucidate the underlying molecular mechanisms and further refine the predictive model.

## Conclusion

The synergistic interaction between FGFR4 and ICs (IL-6, TNF-α, and CRP) significantly contributes to DTC recurrence. The integrated detection model serves as an innovative tool for postoperative risk stratification and personalized therapeutic strategies. These findings provide valuable insights for future clinical evaluation of DTC progression, paving the way for more precise prognostic assessment and targeted interventions.

## Dodatak

### Consent for publication

All participants involved in this study provided explicit consent for the publication of data and results derived from the research.

### Availability of data and materials

The datasets analyzed during the current study are available from the corresponding author upon reasonable request.

### Acknowledgment

Not applicable.

### Conflict of interest statement

All the authors declare that they have no conflict of interest in this work.

### Contributions

Authors made equal contributions in this work.
